# Ionic liquid-assisted electrochemical exfoliation of carbon dots of different size for fluorescent imaging of bacteria by tuning the water fraction in electrolyte

**DOI:** 10.1007/s00604-016-1877-5

**Published:** 2016-07-05

**Authors:** Xuehua Li, Zhiwei Zhao, Chen Pan

**Affiliations:** School of Electronic Science and Engineering, Southeast University, Nanjing, 210096 People’s Republic of China

**Keywords:** Graphite rods, 1-butyl-3-methylimidazolium, Shewanellaoneidensis MR-1, Fluorescence, Decay times, Quantum yield, FTIR, X-ray photoelectron spectroscopy, HRTEM

## Abstract

An electrochemical approach is introduced for synthesis of carbon dots (CDs) by exfoliating graphite rods at a voltage of 15 V in an electrolyte consisting of a mixture of water and two ionic liquids. It is found that the size of the CDs can be tuned by varying the fraction of water in the mixed electrolyte; CDs in sizes of 4.9, 4.1 and 3.1 nm are obtained if the electrolyte contains water in fractions of 24, 38 and 56 %, respectively. The CDs have a quantum yield of almost 10 % and display the typical excitation wavelength-dependent maxima of photoluminescence, strongest at excitation/emission wavelengths of 360/440 nm. Fourier transform infrared and X-ray photoelectron spectroscopy show the CDs to have oxygen functional groups on their surface which strongly improve solubility. The CDs were applied to image cells of the electricity-producing bacteria Shewanellaoneidensis MR-1.

**Electronic supplementary material:**

The online version of this article (doi:10.1007/s00604-016-1877-5) contains supplementary material, which is available to authorized users.

**Graphical Abstract:**
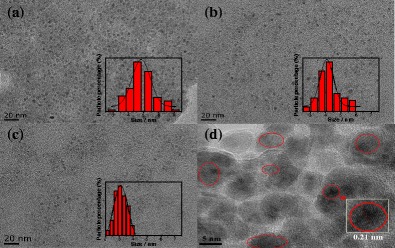
An electrochemical approach is introduced to synthesize carbon dots by exfoliating graphite rods in mixed electrolyte of water and ionic liquids. The increasing size of carbon dots was realized by reducing the volume of water in the mixed electrolyte. The carbon dots were used to fluorescently image the electricity-producing bacterium Shewanellaoneidensis MR-1.

## Introduction

Carbon dots (CDs) have attracted considerable research interest due to their fascinating optical properties, excellent biocompatibility and water solubility [1]. Owing to these unique and novel properties, they have been successfully used for fluorescent probes, photovoltaic devices, photocatalysis, and bioimaging [2–5]. Various approaches have been adopted to synthesize CDs, such as microwave synthesis, ultrasonic treatment, carbon soot, reverse micelles, hydrothermal treatment [6–8]. However, most of these approaches were complex and rigorous processing, which may cause adverse effects to products, such as redundant post-treatment, poorly crystalline impurities and inadequate performance. Electrochemical synthesis of CDs has become popular due to well controllable synthesis at room temperature. Herein, we introduce a method that the ionic liquids (ILs) mixed with different fraction of water was chose as electrolyte and graphite rods were used as electrode and also as carbon source.

ILs have been considered as “green” alternatives to conventionally inorganic solvent due to negligible vapor pressure, wide electrochemical potential window, high ion conductivity, good thermal stabilities [9]. These unique properties render ILs a very useful solvent for electrochemistry electrolyte. The ILs was mixed with different fraction of water as electrolyte. The added water in the electrolyte not only will disrupt the internal organization of ILs and will change the liquid structure by forming hydrogen-boned network, but also will be dissociated into hydroxyl radicals by applying bias voltage. Thus, the content of water in mixed electrolyte may be a key factor to shape and behaviors of the obtained CDs. To the best of our knowledge, there were few related reports on this topic. Hence, the influence of different fraction of water in mixed electrolyte on CDs was investigated and the results were discussed. The clear phenomenon of dependent excitation-wavelength in photoluminescence (PL) spectra was also observed from CDs, and the forming and PL mechanisms of CDs were discussed. Existing bacterial imaging was usually indirect detection of bacterially-secreted metabolites or visualization of bacterial colonies [10, 11]. There is still need of an approach that can directly detect and provide morphological details. As one of pioneers for bacteria bioimaging [12, 13], the exfoliated CDs have been successfully used to label a kind of bacteria of Shewanellaoneidensis MR-1.

## Experimental

### Materials and chemicals

ILs of 1-butyl-3-methylimidazolium tetrafluoroborate ([BMIm][BF4]) and 1-butyl-3-methylimidazolium hexafluorophosphate ([BMIm][PF6]) were purchased from Suzhou Highfine Biotech Co.,Ltd (Suzhou, China, http://highfine.cn.china.cn/) and used without any further purification. The graphite rods were obtained from Sinopharm Chemical Reagent Co.,Ltd (Shanghai, China, http://www.sinoreagent.com/).

### Synthesis of CDs

**Fig. 1:**
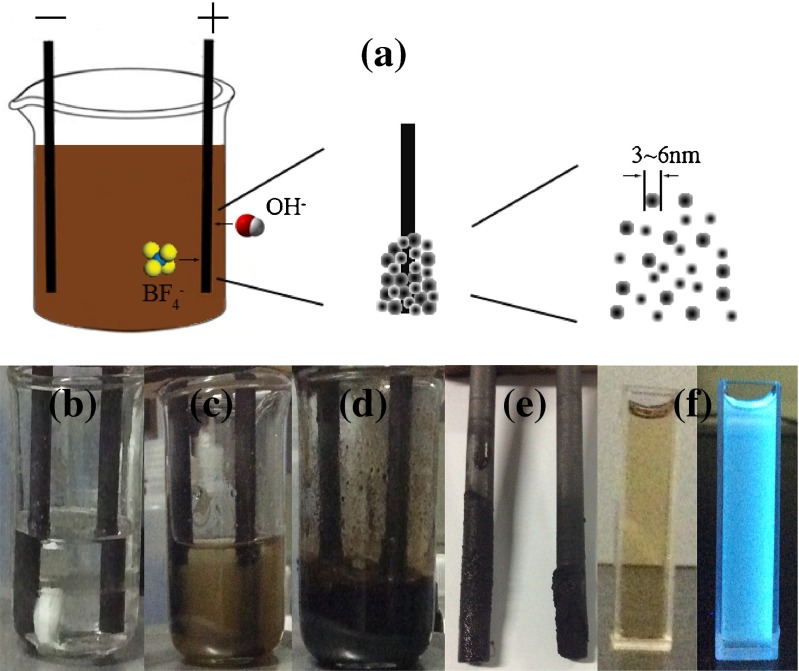
**a** The schematic illustration of the exfoliation process of CDs; **b**–**d** the photographs of the process of exfoliation; **e** the photograph of the exfoliated graphite rods; **f** The photographs of CDs in aqueous solutions under visible (*left*) and UV (*right*) light at wavelength of 365 nm

According to classical two-electrode configuration, two graphite rods were employed as working electrode and counter electrode, respectively, but also as the carbon source of CDs for electrochemical exfoliation. The rods (10 cm in length and 0.6 cm in diameter) were successively rinsed in ethanol and distilled water via ultrasonic treatment for half an hour. After that, the rods were vertically inserted in electrolyte with a parallel separation of 2 cm. The electrolyte consisted of the same volume ILs and different volume of distilled water, in which the ILs consisting of 4 mL 1-butyl-3-methylimidazolium tetrafluoroborate ([BMIm][BF4]) and 4 mL 1-butyl-3-methylimidazolium hexafluorophosphate ([BMIm][PF6]) were mixed with different fraction of water of 2.5, 5 and 10 mL, respectively. The electrochemical exfoliation was carried out by applying static potential of 15 V on the graphite electrodes, supplied by a direct current (DC) power. Throughout the course of experiment, the electrolyte was continuously stirred by a magnetic stirrer to accelerate reaction. After applying potential, distilled water of electrolyte was moderately dissociated into hydrogen and oxygen, releasing in type of bubbles from electrode. The colorless electrolyte gradually changed to yellow, to brown and finally to dark brown (shown in Fig. 1b–d). The obtained dark brown dense electrolyte contained a large number of CDs,which were needed to be extracted from the electrolyte. The electrolyte was mixed with some distilled water. Then it was treated using ultrasonic wave for 30 min to partly transfer the CDs in the electrolyte into water. At last, it was centrifuged at 10000 rpm (7000 g) for the separation of supernatant from ILs. The obtained supernatant contained many CDs and were denoted as CDs-W-2.5, CDs-W-5, and CDs-W-10, respectively, according to the different fraction of water in electrolyte.

### Characterization

Transmission electron microscopy (TEM) and high-resolution TEM (HRTEM) images were taken using a JEM-2100transmission electron microscope (Jeol, Japan). The normal TEM samples were prepared by dropping the aqueous solution of CDs onto copper grids and then dried under drying lamp. The statistical size distribution of CDs was obtained using the software of Nano Measurer 1.2 on the basis of TEM images (counting more than 200 dots for each sample in different images). Fourier transform infrared spectroscopy (FTIR) patterns were measured in range of 400–4000 cm^−1^ on a Nicolet 5700 FTIR spectrophotometer(Nicolet, USA). The X-ray photoelectron spectra (XPS) were recorded on a PHI 5000 Versa Probe electron energy spectrometer (UIVAC-PHI,Japan). Light absorption properties were obtained using ultraviolet–visible (UV–vis) spectrophotometer (UV-3600,Shimadzu, Japan). The measurement of PL was carried out on a Fluoromax-4 fluorescence spectrophotometer (Horiba, Japan). Fluorescence lifetime was measured with a time-resolved spectroscope FluoroLog 3-TCSPC (Horbia, Japan). The fluorescence microscope images were taken with a Leica TCS SP5 confocal microscope (Leica Microsystems, Germany).

### Quantum yield (QY) measurement

The quantum yield of CDs was achieved by using a comparative method (quinine sulfate as standard sample) using the following equation^19^:

$\upphi ={\upphi}_q\times \frac{I}{I_q}\times \frac{A_q}{A}\times \frac{n^2}{n_q^2}$ where, the subscript *q* denotes quinine sulfate, Φ stands for the QY, *I* designates the integrated emission intensity, *n* and *A* are the refractive index and optical absorbance, respectively. Quinine sulfate (QY: 54 % excited at 360 nm) was dissolvssed in 0.1 M H_2_SO_4_ with refractive index of 1.33 and aqueous solution of CDs has the same value of refractive index. In order to minimize interference, both individual absorbance of quinine sulfate and CDs were adjusted down to 5 % at the excitation wavelength of 360 nm.

### Bacteria imaging experiments

The bacteria of *Shewanellaoneidensis MR-1* were seeded and incubated in medium that contained 10 g⋅L^−1^ NaCl, 10 g⋅L^−1^ Casein Tryptone and 5 g⋅L^−1^ Yeast extract at room temperature for 2 days. After extracting the bacteria using centrifugal machine and then rinsing with phosphate buffer, the bacteria were further cultured in 10 mL fresh LB containing aqueous solution of CDs at room temperature for 5 days. Finally, the bacteria were washed and then imaged using a Leica TCS SP5 confocal microscope.

## Results and discussion

**Fig. 2:**
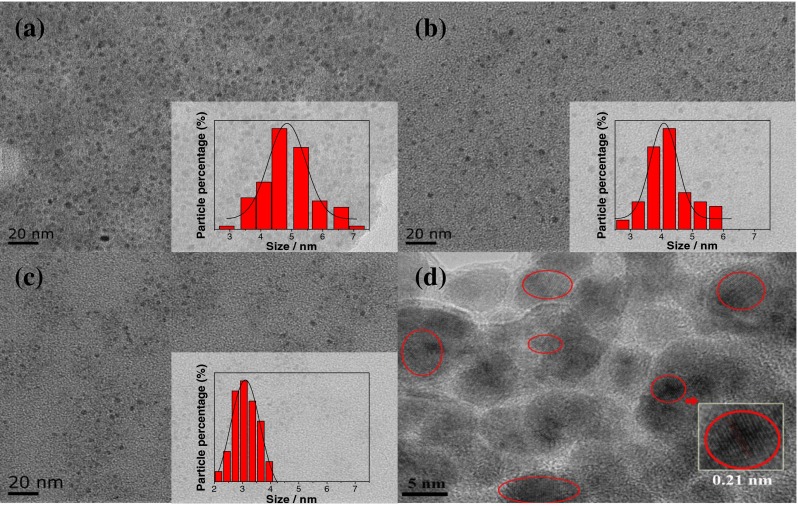
TEM images of CDs synthesized by electrochemical exfoliation at different fraction of water: CDs-W-2.5, CDs-W-5 and CDs-W-10 corresponding to (**a**) (**b**) and (**c**), respectively. The insets showed corresponding size distribution and fitted Gaussian curves. HRTEM image of CDs-W-2.5 synthesized was shown in (**d**)

Figure 2a, b and c show the TEM images of CDs-W-2.5, CDs-W-5 and CDs-W-10, respectively. The statistical analyses about the sizes of CDs give a fitting Gaussian distribution were also shown in corresponding insets. It can be easily found that these CDs were monodispersed and quasi-spheres in shape, well agreed with previous reports [14, 15]. Interestingly, from the center values of all fitting Gaussian curves as shown in insets, it can be identified that the sizes of CDs-W-2.5, CDs-W-5 and CDs-W-10 decreased as the increasing volume of water, with the size of 4.9, 4.1 and 3.1 nm, respectively. The CDs with different sizes have been successfully fabricated. For example, Hu et al. accomplished size tailoring of CDs by changing the laser pulse widths in laser synthesis [16]. Rhee et al. have synthesized CDs with size tunability by tuning water-surfactant molar ration, employing reverse micelles as nanoreactors in carbonization of sugar [17]. Comparing with these methods, our approach was not so stringent experimental conditions and was easy to implement. The HRTEM image of CDs-W-2.5 was selected as a typical representative of all HRTEM images and shown in Fig. 2d. It was clearly observed that CDs possessed distinct crystal lattice with lattice spacing around 0.21 nm, agreeing well with the (100) lattice plane of graphite [18–20], which showed an sufficient evidence that the CDs were carbon genic materials.

Mechanistically, the process of CDs’ exfoliation from graphite rods can be illustrated by Fig. 1a. After applying bias voltage beyond decomposition voltage, water in the electrolyte was firstly decomposed into hydroxyl (−OH) and oxygen (−O) on the electrode. Imidazaolium cations ([R_1_R_3_I_m_]^+^) and anions of BF_4_^−^ and PF_6_^−^ also separately moved toward cathode and anode in electric field, respectively. All of these charged ions were able to attack and disrupt electrode, leading to corrosion on the surface of the rods. Moreover, due to capturing free electron from DC power supply, the imidazolium cations of ILs were reduced into imidazolium free radicals at the electrode. This reduction process may partly occur in the corrosion of disrupted surface. Thus, these bigger imidazolium free radicals can be created in interlayer of graphite and be inserted into π bonds of graphite plane. Subsequently, due to oxidation and hydroxylation by these radicals and corrosion from attacking of charged ion near electrode, defects of sites and boundaries in the graphite rods opened up initially. Then, the bigger ones can intercalate into interlayer of graphite from this opening up place. Two kinds of bigger radicals containing imidazaolium radicals and depolarization of anions mainly finished this process, leading to expansion of interlayer distance of graphite and the exfoliation of CDs from graphite rods. Based on above analysis, it is reasonably speculated that, during the process of exfoliation, more water in electrolyte means more time will be consumed for decomposition, lead to more charged ions attacking to bigger nanoparticles or CDs exfoliated from rods. These bigger ones were further split into smaller CDs due to suffering from more attacking. Hence, more water existed in electrolyte, more smaller CDs would appear in the obtained solution.

**Fig. 3:**
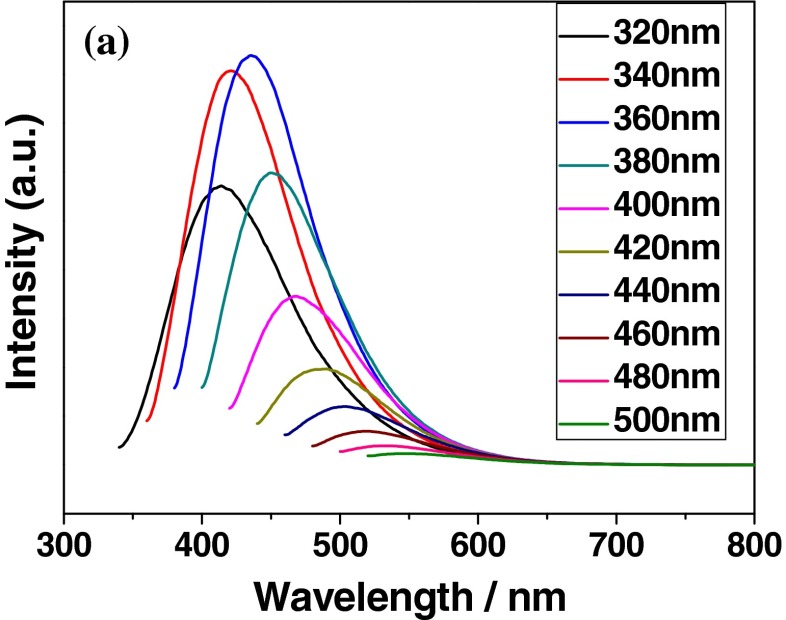
PL spectra of the CDs-W-2.5 excited at different wavelengths

As the most important property of CDs, the PL emission spectra of CDs excited at different excitation wavelengths were recorded and shown in Fig. 3. The obvious excitation-dependent red-shift PL behaviors for emission maximum were observed from PL spectra of CDs. As the excitation wavelength increased from 320 to 500 nm, the emission band maximum shifted to longer wavelength from 414 (violet) to 548 (green) nm,which was well in agreement with previous reports [1, 21, 22]. The solution of CDs showed the strongest emission peak locating at 439 nm excited by 360 nm and had strong intensity in range of 375 to 525 nm. These results were enough evidence of an interesting and common dependence on the excitation wavelength for the CDs. Moreover, bright blue PL emission of the solution CDs was strong enough to be easily observed with bare eye, (shown in photograph of Fig. 1f), upon the irradiation of 365 nm by handy UV lamp. By selecting quinine sulfate as standard reference and 360 nm as excitation wavelength, the QY of CDs were measured and calculated to be nearly 10 %. Up to date, the exact origin of excitation-dependent PL behaviors are still not fully understood, which has been probably due to differently sized nanoparticles, different emissive trap states on the surface of CDs or other unresolved mechanisms. In order to further investigate the PL property, we compared PL behaviors of CDs with different sizes on the same excitation wavelength, and it did not show the size-dependent red-shift (shown in supporting information, Fig. S1). The size-dependent red-shift is common behavior for quantum dots (QDs) and has been widely accepted to be results of quantum size effect. The size-independent phenomenon of CDs proves that the quantum size effect is not the main derivation of PL behavior for the CDs.

The UV–vis absorption spectra of CDs were measured and shown in supporting information (Fig. S2). These curves of absorption showed three obvious features: with similar shapes in range of 250–800 nm, two shoulder peaks centering around 270 and 320 nm [23] and a broad tail extending over visible range. The similarity in absorption shape may be attributed to similar structure and surface state of CDs. The peak appearing nearly at 270 nm is ascribed to the π-π* transition of C = C [24], while the latter indicated the *n*-π* transition of C = O bond [25].

**Fig. 4:**
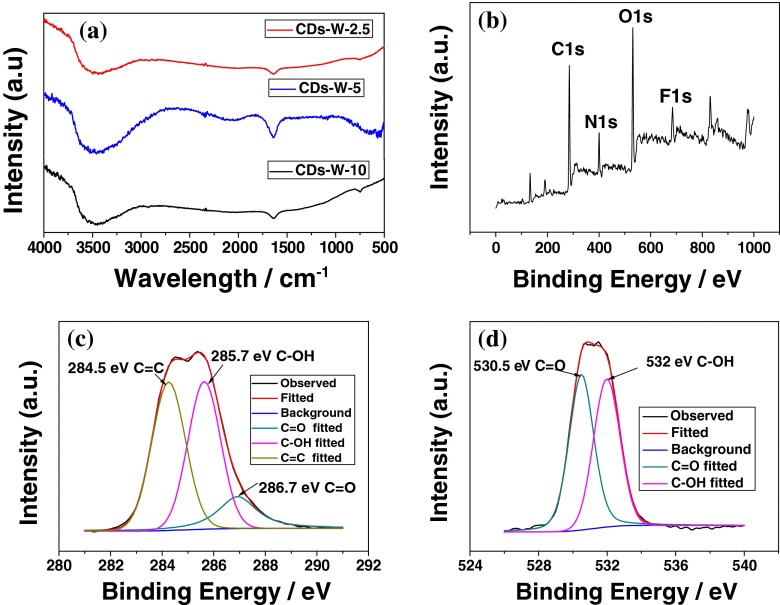
**a** FTIR spectra of CDs-W-2.5, CDs-W-5 and CDs-W-10; **b** XPS patterns of the CDs-W-2.5; **c**, **d** High-resolution XPS spectra of the C 1 s and O 1 s peaks of CDs-W-2.5, respectively

In order to understand components and chemical structures of CDs, FTIR spectra and XPS spectra were characterized and the results were shown in Fig. 4. These FTIR spectra of the CDs (Fig. 4a) with different sizes exhibited a similar shape as well as peak positions, suggesting all samples with similar chemical composition and structure. The typical peak at 3458 cm^−1^ was associated with the stretching vibration and in-plane bending vibration of –OH [21, 26], and the hydroxyl came from the decomposition of water in electrolyte. The band of 1637 cm^−1^ was present evidence of aromatic C = C [27]. The most distinct and the strongest intensity in all peaks were the characteristic absorption bands of hydroxyl. The peaks in spectra corresponding to oxygen-containing groups and other functional groups indicated successful oxidation of graphite and the formation of functional groups.

The XPS spectra of CDs was measured and shown in Fig. 4b–d. The XPS survey spectrum showed two predominant peaks of C1s centered at 285 eV and O1s centered at 531 eV, respectively, indicating that the CDs mainly contain carbon and oxygen elements. There were also some weaker P 2p, B, N 1 s and F peaks, which may be attributed to the residuals of ILs on CDs. The C1s core level peak, as shown in Fig. 4c, can be resolved into three components centered at 284.5, 285.5 and 287.4 eV, respectively, corresponding to C = C, C-OH and C = O bonds [28], which was also evidenced by FTIR. These -OH and C = O bonds may originate from the binding of dangling carbon bond on surface of exfoliated carbon fragments and hydroxyl radicals from decomposition of water. As the other major element, high-resolution spectrum of O1s was recorded and shown in Fig. 4d. The fitted curves was deconvoluted into two peaks at 530.5 and 532 eV, which can be assigned to C = O and C–OH, respectively [29].

The time-resolved fluorescence decay curves of CDs were measured by single photon counting method at 360 nm excitation and 440 nm emission (as shown in Fig. S3). The three curves with similar shape showed a few differences on radian in range of 10 to 25 ns. These decay lifetimes can be well fitted to a triple-exponential function with average lifetimes of 8.0, 7.0, and 5.9 ns corresponding to CDs-W-2.5, CDs-W-5 and CDs-W-10, respectively. The diverse fluorophores or energy levels presented in CDs may be responsible for these multiple lifetimes.

Although a large number of approaches have been developed to synthesize CDs relevant to PL emission and much progress have been made to improve its property of PL, there are still not widely accepted consensus on origins of PL from CDs. Several mechanisms have been hypothesized for explanation of PL from CDs mainly including quantum size effect and defect traps (surface energy traps) [30, 31]. The size-independent PL behavior (as shown in Fig. S1) revealed that the quantum size effect played no or a small role in PL mechanism. However, based on results of FTIR and XPS, it has been verified that a large amount of functional groups had been attached to the CDs by binding dangling bond or other effect. Comparing to graphite bulk, these functional groups can be considered as defect traps. These defects can increase opportunity of facilitating the trapping of photoinduced electrons and holes, more like to the surface energy trap. The PL emission of the CDs may be a result of this kind radiative recombination of electrons and holes trapped by these surface energy traps [32]. Besides, the very small size (probably sub-5 nm) can create large surface-to-volume ratio, also leading to increase of surface energy traps [1]. It has been evidenced that a lot of -OH groups attached on surface of CDs from results of FTIR and XPS. Hydroxyl has been regarded as a kind of electron donator in CDs [33], which are of great benefits to increase the opportunity of recombination of electrons and holes.

**Fig. 5:**
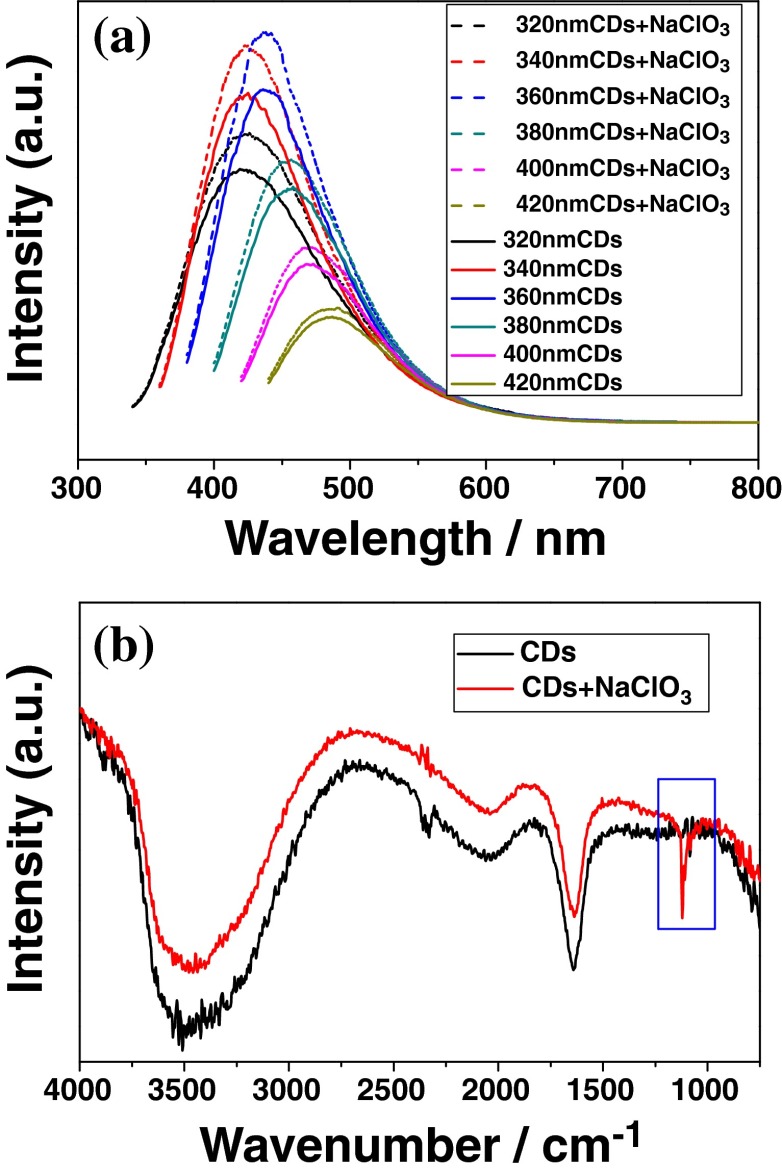
PL spectra (**a**) and FTIR spectra (**b**) of CDs and CDs + NaClO_3_

To clarify this hypothesis, the experiments of CDs oxidized by NaClO_3_ (donated as) were implemented. The comparison of PL and FTIR spectra of them were shown in Fig. 5. The PL emission compared spectra of CDs and CDs + NaClO_3_ are shown in the Fig. 5a, whose excitation wavelength increased from 320 to 420 nm with the increment of 20 nm. It was obviously observed that PL intensities of CDs + NaClO_3_ have increased to some degree at each excitation wavelength. However, the position of each emission peaks remained stable, indicating that both of them possessed similar PL mechanism. In FTIR comparison of CDs and CDs + NaClO_3_ (shown in Fig. 5b), the most distinct change was the enhancement of absorption band at 1118 cm^−1^ corresponding to the stretching vibration bands of C-O attributed to the oxidation of NaClO_3_ [34]. During this oxidation process, CDs + NaClO_3_ were further added more king of defects on surface of CDs + NaClO_3_ due to the appearance of C-O, which favored to trap more electrons and holes. The nearly coincident time-resolved fluorescence decay curves of CDs and CDs + NaClO_3_ fit a triple-exponential function with similar average lifetimes of 8.0 and 8.1 ns, respectively (shown in Fig. S4). This indicates that both of them have similar decay mechanism or emission mechanism. Based on above analysis, it can be concluded that the oxidation of CDs by NaClO_3_ increased the amount of defect traps which facilitated more radiative recombination of electron and holes, leading to the enhancement of PL intensity. However, this process only enhanced the intensity of PL spectra, and it did not change the position of PL emission peaks and mechanism.

**Fig. 6:**
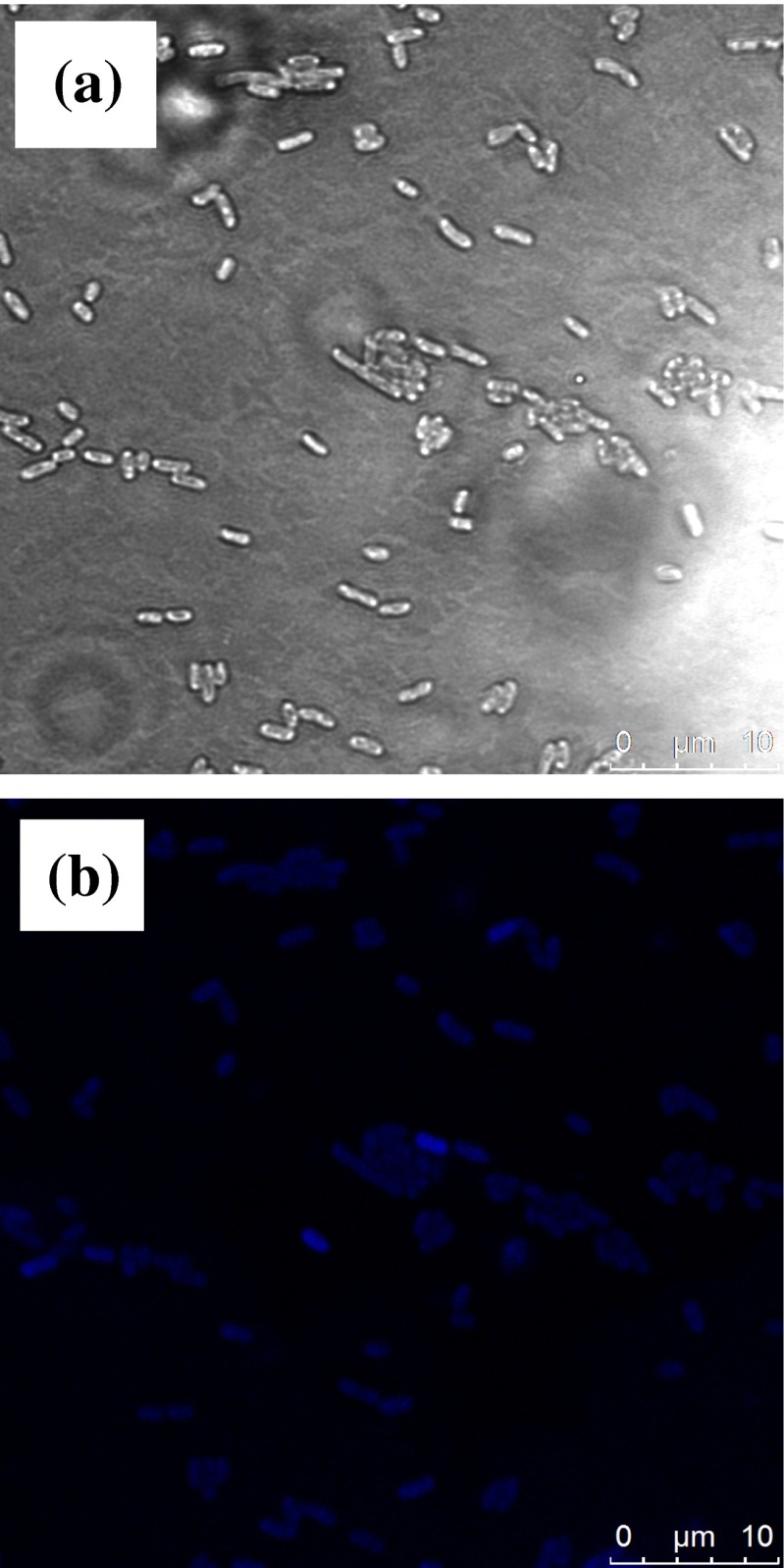
Bright field (**a**) and fluorescence microscope images (**b**) of Shewanellaoneidensis MR-1 incubated with the solution of CDs

As the most important application, CDs have been widely investigated and utilized in the bioimaging or biological labeling. However, to the best of our knowledge, most of bioimaging experiments were carried out using various cancer cells, and there are few studies using bacteria as labeled specimen [3, 35]. Existing bacterial imaging was usually indirect detection of bacterially-secreted metabolites or visualization of bacterial colonies. There is still need of an approach that can directly detect and provide morphological details. *Shewanellaoneidensis MR-1*, which is a kind of bacteria with ability of reducing metal to generate electricity, was firstly selected for bioimaging under in vitro conditions. Figure 6 shows the bright field and laser confocal microscopy images of *Shewanellaoneidensis MR-1* labeled with CDs excited at 405 nm. It is readily seen that the Shewanellaoneidensis MR-1 incubated with CDs became bright excited at 405 nm, whereas the controlled bacteria shown nearly no visible fluorescence detected under the same conditions. Besides, we also took images at other wavelength of excitation of 488 and 638 nm and obtained green (542 nm) and red (658 nm) images (shown in Fig. S5), respectively. Exciting at three different wavelengths, the bacteria incubated with CDs showed three different colors, which was an evidence of eliminating autofluorescence. There was obvious and bright difference between bacteria and background in the higher resolution images with single bacteria. These evidenced that the CDs have been internalized by the bacteria. Based on the above analysis, it can be easily concluded that CDs may be used for not only cell labeling but bacteria labeling in vitro conditions. There is still a limitation that the CDs have a relatively low QY, which influence the brightness of bacterial images (as shown in Fig. 6 and Fig. S5). A lot of work was needed to be done for improvement of brightness.

## Conclusions

In summary, size-decrease CDs were synthesized by increasing fraction of water in electrolyte using an electrochemical exfoliation from graphite rods. The analysis of components and chemical structures of CDs have proved that a large number of functional groups appeared on surface of CDs. These groups added more defects on surface of CDs leading to more recombination of electrons and holes. Based on these results, the origin of PL may be attributed to the radiative recombination of surface-trapped electrons and holes. CDs oxidized by NaClO_3_ showed enhanced PL intensity due to the increase of groups. The CDs can be successfully used to label bacteria from luminescence bioimaging in Shewanellaoneidensis MR-1. There is still a main limitation that the CDs have relatively low QY of 10 %, which must be enhanced in the future work.

## Electronic supplementary material

Below is the link to the electronic supplementary material.

ESM 1 (DOCX 1695 kb)
