# Supplementary material for: Ionic liquid-assisted electrochemical exfoliation of carbon dots of different size for fluorescent imaging of bacteria by tuning the water fraction in electrolyte
Source: Mikrochim Acta. 2016 Jul 5;183:2525–32. doi: 10.1007/s00604-016-1877-5 (PMC4977338; doi:10.1007/s00604-016-1877-5)
Supplement: Supplementary file 1 — (DOCX 1695 kb) [file 604_2016_1877_MOESM1_ESM.docx]

**Electronic Supplementary Material**

**Ionic liquid-assisted electrochemical exfoliation of carbon dots of different size for fluorescent imaging of bacteria by tuning the water fraction in electrolyte**

Xuehua Li， Zhiwei Zhao^^[[1]](#footnote-1)^*^, Chen Pan

*School of Electronic Science and Engineering, Southeast University, Nanjing 210096, P.R.China*

Fig.S1 Normalized PL spectra of CDs-2.5, CDs-5 and CDs-10, respectively, excited by the same wavelength of 360 nm

Fig. S2. The UV-vis absorption spectra of CDs-2.5, CDs-5 and CDs-10, respectively.

Fig. S3 The time-resolved fluorescence-decay curve of CDs (ex at 360 nm) measured at 440 nm. The instrument response function (IRF) is also listed for comparison.

Fig. S4 The time-resolved fluorescence-decay curve of CDs and CDs+NaClO_3_;


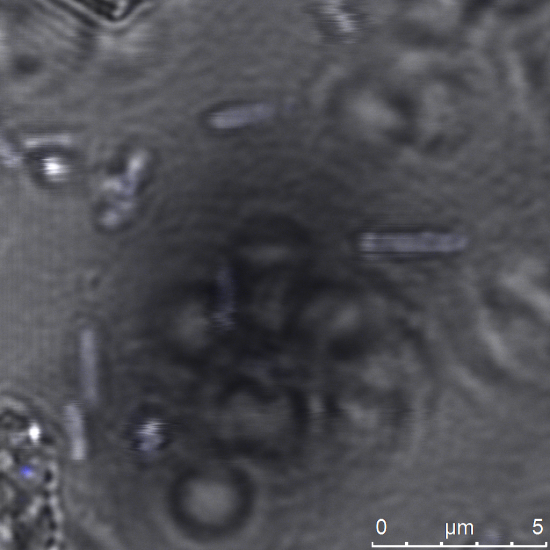

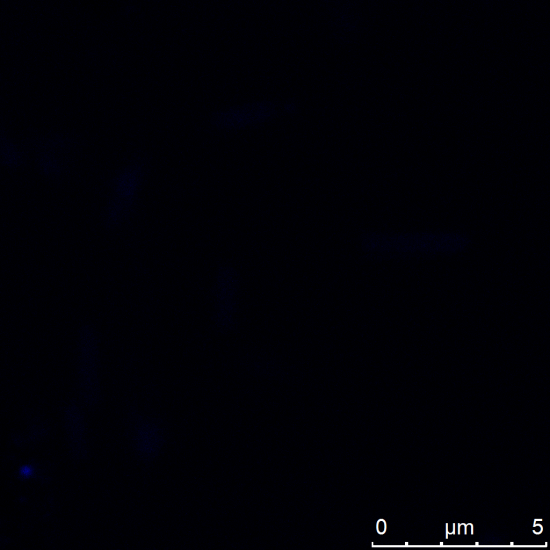

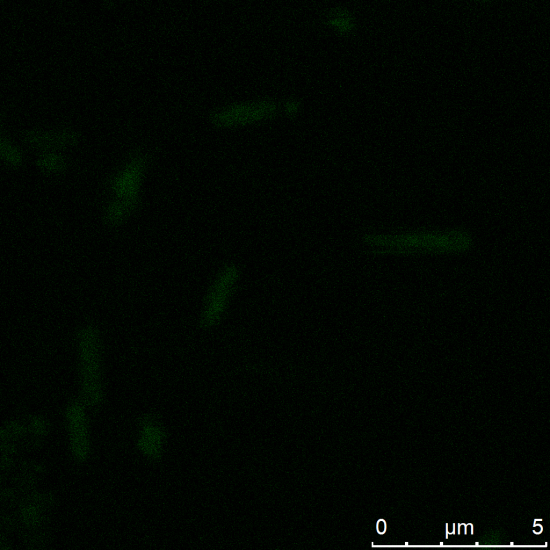

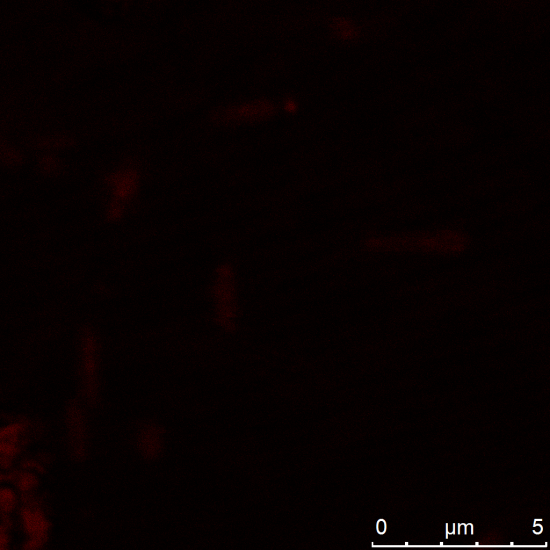


Fig. S5 Bright field and fluorescence microscope images of Shewanellaoneidensis MR-1 incubated with the solution of CDs.

Fig.S6 The influence of the pH value on CDs-2.5 fluorescence (excited at 360 nm).

1. * Corresponding author Email address: [Zhao_zw@seu.edu.cn](mailto:Zhao_zw@seu.edu.cn); Tel: +086-025-83792250; Fax: 086-025-83793222 [↑](#footnote-ref-1)
